# Dysphagia After Prolonged Intubation in SARS-CoV-19 Patients: A Single Institution Retrospective Review

**DOI:** 10.7759/cureus.41544

**Published:** 2023-07-07

**Authors:** Elycia Kazemian, Mark Solinski, Amy Wozniak, Steven Charous

**Affiliations:** 1 Otolaryngology - Head and Neck Surgery, Loyola University Medical Center, Maywood, USA; 2 Statistics, Loyola University Chicago Health Sciences Division Center for Translational Research and Education, Maywood, USA

**Keywords:** sars-cov-2, swallow, prolonged intubation, covid-19, functional dysphagia

## Abstract

Objective: To determine the impact of various factors on swallowing in SARS-CoV-19 patients after prolonged intubation.

Methods: A retrospective chart review of SARS-CoV-19 patients intubated between February 2020 and March 2021 was performed. Independent variables, including duration and factors of intubation, and patient demographic characteristics were analyzed. Formal swallow studies were performed for patients who failed a screening swallow evaluation.

Results: Seventy-three individuals of 308 patients reviewed had a dysphagia score of ≤5. A total of 49% of patients’ dysphagia resolved prior to discharge, with a median of eight days between extubation and the last evaluation. The median duration of intubation was 11 days. Increasing age, congestive heart failure, cerebrovascular disease, and hypertension were associated with dysphagia at the first and/or last evaluation. Hispanic ethnicity was associated with a decreased risk of dysphagia (all p<0.05).

Conclusions: Although various patient factors including age and congestive heart failure were associated with the development of dysphagia after prolonged intubation, the length of intubation was not.

## Introduction

Orotracheal intubation (OTI) provides access to ventilatory assistance in anesthetized or mechanically ventilated patients [1]. OTI is subcategorized into short- and long-term duration. Intubation lasting longer than 48 hours is considered long-term and anything less is considered short-term [2]. Oropharyngeal dysphagia is defined as an abnormality in the upper aerodigestive tract that alters the swallowing mechanism [3]. In general, the literature strongly supports the association between prolonged intubation and subjective and objective dysphagia. Frajkova et al. list multiple mechanisms that contribute to the development of dysphagia, including laryngeal and oropharyngeal trauma, neuromuscular weakness, altered sensorium, and impaired synchronization of breathing and swallowing [4]. Effects on swallowing have been seen after just 48 hours of endotracheal intubation [5].

Patients experiencing dysphagia are more likely to have a delayed return to normal diet, aspiration, and infection, any of which can lead to delayed hospital discharge [6]. With this in mind, the timing of tracheostomy has been an important team decision in intensive care units (ICUs) around the world. Tracheostomies in this setting are performed for a multitude of reasons, including to decrease post-extubation dysphonia, dysphagia, subglottic and glottic stenosis; to facilitate weaning from the ventilator; and to improve patient comfort. A recent systematic review proposed that tracheostomy should be performed within seven days of intubation in critically ill patients, but a study linking aspiration to prolonged intubation was unable to identify a true link between the length of intubation and dysphagia [7,8]. In today’s unprecedented pandemic medical environment, the recommendations to perform tracheostomy within seven days have been relaxed for a variety of reasons, mainly due to the decreased survival rates of critically ill SARS-CoV-2 (Covid-19) patients and the high transmission risk to the surgeon and other healthcare providers. In a study by Ferri and colleagues, data showed that tracheostomy did not significantly alter the prognosis of SARS-CoV-2 patients and that the indication for tracheostomy should be considered after 20 days of endotracheal intubation in an effort to reduce the risk of contagion to involved healthcare workers [9]. Our institution followed a similar timeline during the early SARS-CoV-2 pandemic; patients were considered candidates for tracheostomy after three weeks of endotracheal intubation and only in the setting of clinical stability or improvement.

Given that historically the duration of endotracheal intubation did not extend to such lengths because of the well-known aerodigestive complications, the opportunity to investigate the effects of extended intubation on oropharyngeal dysphagia in the Covid-19 population was unique. In addition, the fact that the overall underlying etiology of the intubation was the same for these patients allowed for the investigation of other independent variables in a more pure manner. It is beneficial to understand what independent variables predispose patients to dysphagia after prolonged intubation. We hypothesized that the duration of intubations would correlate with increased dysphagia and that age, size of the endotracheal tube (ETT), and diabetes may also have a negative impact on post-extubation swallowing.

## Materials and methods

Our study is a retrospective chart review of patients with a history of OTI due to Covid-19 respiratory illness between February 2020 and March 2021 approved by the Institutional Review Board (LU214062). Data was collected from review of the electronic medical record system, including age, sex, race, ethnicity, admission date, discharge date, discharge disposition, date of last positive lab result, body mass index, ICU stay, length of ICU stay, ventilator use, date of intubation and extubation, ETT size, dates of speech-language pathology (SLP) evaluation, swallow scores at first and last SLP evaluation, tracheostomy placement, and various comorbid medical conditions. Inclusion criteria were positive Covid-19 lab result, hospital stay >23 hours, age >18 years, and endotracheal intubation. Exclusion criteria were prior diagnosis of dysphagia, death, and neurologic disorders (i.e., stroke).

All extubated patients receive a bedside swallow screening by a nurse, which involves an assessment of the patient's general readiness for oral intake (i.e., mental status, level of alertness, strength, etc.) followed by a screening with small sips of water. If the patient has any signs of concern, such as choking, wet voice, or oral incompetence, a formal evaluation by SLP is done. If a patient failed this screening, swallowing was evaluated by SLP using the National Outcomes Measurement System Functional Communication Measures scale from the American Speech and Hearing Association (2012) [10]. This scale describes a patient’s swallowing ability and is generally used to track progress over time. The scale consists of seven levels, ranging from inability to swallow (level 1) to normal swallowing abilities (level 7). These scores dictate the diet recommendation, ranging from NPO to full general diet, respectively. For our study, scores on this scale were grouped, with a score of ≤5 identified as dysphagia and a score of ≥6 identified as normal swallowing. If a patient had more than one evaluation by SLP, the first evaluation was used as time 1, and the last recorded evaluation during the hospital stay was used as time 2.

Frequency and column percentages are presented for categorical patient factors; median (Q1, Q3) are presented for continuous patient factors. If a patient had an initial swallow assessment and was deemed no-dysphagia and was missing a second SLP evaluation, the second assessment was imputed as "no dysphagia." Of patients who were not terminally extubated and not assessed by an SLP, outcomes were imputed as ‘no dysphagia’ at both time points given that lack of escalation of care to SLP assumes no difficulties with oral intake. Univariable logistic regression models were used to estimate associations of patient factors with dysphagia at time 1 and dysphagia at time 2; multivariable logistic regression was used to estimate the adjusted associations of risk factors significant at the univariable p<0.05 level. In both analyses, a sensitivity analysis was performed using only those with an SLP to compare qualitative results between imputation and no-imputation populations. In the subset of patients with two swallow assessments, univariable logistic regression was used to estimate associations of risk factors with an increase in swallow score from time 1 to time 2. No imputations were made for this analysis. All analyses were performed using SAS 9.4 (Cary, NC); p-values were 2-sided and p<0.05 were deemed statistically significant. 

## Results

There were 308 patients who were intubated due to severe Covid-19 respiratory illness between February 2, 2020, and March 1, 2021. A total of 80 patients were terminally extubated and excluded from statistical analyses. These patients tended to be older, male, and diabetic. The remaining 228 patients included in our analysis are composed of 94 patients with at least one post-extubation swallow evaluation and 134 patients who did not have a formal swallow evaluation but were considered "no dysphagia" (Table 1).

**Table 1:** Cohort Summaries Missing in SLP group: 19 ETT, 5 mech vent, 21 swallow 2, 6 dysphagia at time 2 (15 imputed as non-dysphagia, 6 unable to impute) Missing in no SLP group: 45 ETT, 40 mech vent Missing in terminally extubated, no SLP group: 20 ETT, 18 mech vent "-" indicates not applicable or all missing SLP, speech language pathology; BMI, body mass index; CVD, cardiovascular disease; COPD, chronic obstructive pulmonary disease; CHF, chronic heart failure; MI, myocardial infarction; ETT, endotracheal tube; mech vent, mechanical ventilation; LOS, length of stay

Patient Characteristics	1+ SLP Eval N=94	No SLP Eval (Imputed as No Dysphagia) N=134	Terminally Extubated, no SLP Eval (Excluded from Analyses) N=80
Age, Median (Q1, Q3)	62 (49, 71)	56.5 (43, 67)	67 (60.5, 74.5)
Sex, Male, n (%)	56 (60)	81 (60)	53 (66)
Race, n (%)			
White	45 (48)	69 (51)	39 (49)
Black	26 (28)	18 (13)	14 (18)
Other	23 (24)	47 (35)	27 (34)
Ethnicity, Hispanic, n (%)	41 (44)	91 (68)	46 (58)
BMI, Median (Q1, Q3)	31.7 (27.3, 37.6)	30.8 (25.8, 38.6)	30.9 (25.6, 34.9)
Comorbidity, n (%)			
Cardiac Arrythmias	55 (59)	62 (46)	46 (58)
CVD	18 (19)	21 (16)	9 (11)
COPD	16 (17)	20 (15)	11 (14)
CHF	26 (28)	16 (12)	9 (11)
Diabetes	26 (28)	37 (28)	26 (33)
Hypertension	42 (45)	45 (34)	34 (43)
MI	14 (15)	11 (8)	12 (15)
Obesity	19 (20)	19 (14)	12 (15)
Renal Disease	18 (19)	19 (14)	8 (10)
In-Hospital Characteristics			
ETT Size, n (%)			
<7.5	11 (15)	11 (12)	5 (8)
7.5	34 (45)	51 (57)	35 (58)
>7.5	30 (40)	27 (30)	20 (33)
ETT: Height Ratio, Median (Q1, Q3)	0.1 (0.1, 0.1)	0.12 (0.11, 0.12)	0.11 (0.10, 0.12)
Mech Vent Duration, Median (Q1, Q3)	11 (6, 18)	11.5 (4, 20)	7.5 (4, 16)
LOS, Median (Q1, Q3)	17.5 (9.5, 27.5)	14.5 (9, 24)	11 (6, 17.5)
Discharge Location, n (%)			
Home	26 (28)	31 (23)	-
Nursing	29 (31)	16 (12)	-
Discharge/Transfer/Other	39 (41)	87 (65)	-
Swallow Characteristics			
Swallow 1, Median (Q1, Q3)	4 (1, 5)	-	-
Swallow 1 ≤5, Dysphagia, n (%)	73 (78)	0 (0)	-
Time to Swallow 1, Median (Q1, Q3)	1 (1, 2)	-	-
Swallow 2, Median (Q1, Q3)	7 (5,7)	-	-
Swallow 2 ≤5, Dysphagia, n (%)	31 (35)	0 (0)	-
Time to Swallow 2, Median (Q1, Q3)	8 (5, 12)	-	-
Time from Swallow 1 to Swallow 2, Median (Q1, Q3)	6 (4, 10)	-	-
Difference in Swallow 2-Swallow 1, Median (Q1, Q3)	2 (1, 4)	-	-

Of those with a swallow score (N=94), 73 individuals had a swallow score ≤5 indicating dysphagia at the first evaluation (swallow 1), composing 32% of our patient population and 77.6% of all patients evaluated by SLP. The median time of evaluation was one day post-extubation. Of the 73 patients with dysphagia at the first evaluation, 67 received at least one other SLP evaluation with a median time of six days between the first and last evaluation. 36 recovered and did not have dysphagia at the second evaluation. 31 still had dysphagia at the second evaluation. Those who were not evaluated by SLP and considered non-dysphagia (N=134) were younger and had a higher prevalence of Hispanic ethnicity. This group also had a lower incidence of comorbid conditions, with the exception of diabetes mellitus. Intubation duration was similar between both groups (Table 1).

Of the 31 patients with persistent dysphagia at the time of the last SLP evaluation (swallow 2), 19 had no further follow-up in the chart two years or more after admission, five were deceased or discharged to home hospice, two had continued dysphagia proven by documentation in subsequent admissions or visits, and four had documentation of return to normal swallowing. One patient had long-term dysphagia related to developmental delay. The 26 living patients with dysphagia at the last SLP evaluation were contacted via telephone to obtain long-term follow-up. One additional patient was deceased. 22 were unable to be reached after three attempts to be contacted by telephone. The three patients able to be contacted reported no further problems, with an EAT-10 score of 0, 0, and 2. None of these had seen an SLP for their dysphagia at another institution.

The patients with persistent dysphagia at swallow 2 comprised 13.6% of the entire patient population and 33% of patients evaluated by SLP. Six patients with dysphagia on the initial evaluation did not have a repeat evaluation. The median time between extubation and the last recorded SLP evaluation was eight days. Of the 94 patients with at least one swallow evaluation, one patient was terminally extubated after re-intubation. Eight patients had a tracheostomy placed after endotracheal intubation.

In univariable analysis, we found increasing age and comorbidities of CHF and hypertension to be significantly associated with an increased risk of dysphagia, while Hispanic ethnicity was associated with decreased odds of dysphagia. In multivariable analysis, only increasing age was associated with increased risk of dysphagia (OR (95% CI): 1.02 (1.00, 1.05)) and Hispanic ethnicity was associated with decreased risk of dysphagia (OR (95% CI): 0.47 (0.26, 0.86)) (Table 2). Results were qualitatively similar in the sensitivity analysis using only those with an SLP evaluation.

**Table 2:** Associations with Dysphagia at Time 1 *Per unit increase for age (years), mech vent (days), and time to swallow 1 (days).  Per 0.01 unit increase for ETT: height ratio. **Calculated using univariable logistic regression using complete case analysis. See Table 1 for missingness. ***Calculated using multivariable logistic regression using all variables presented in the column. BMI, body mass index; CVD, cardiovascular disease; COPD, chronic obstructive pulmonary disease; CHF, chronic heart failure; MI, myocardial infarction; ETT, endotracheal tube; mech vent, mechanical ventilation

Patient Characteristics	Median (Q1, Q3) No Dysphagia, N=155	Median (Q1, Q3) Dysphagia, N=73	Univariable Odds Ratio* (95% Confidence Interval)	P-Value**	Multivariable Odds Ratio* (95% Confidence Interval)	P-Value***
Age, Median (Q1, Q3)	56 (42, 67)	64 (53, 72)	1.03 (1.01, 1.05)	0.0014	1.02 (1.00, 1.05)	0.0183
Sex, Male, n (%)	92 (59)	45 (62)	1.1 (0.62, 1.95)	0.7420		
Race, n (%)						
White	78 (50)	36 (49)	Reference			
Black	26 (17)	18 (25)	1.5 (0.73, 3.08)	0.2691		
Other	51 (33)	19 (26)	0.81 (0.42, 1.56)	0.5237		
Ethnicity, Hispanic, n (%)	100 (66)	32 (44)	0.4 (0.22, 0.71)	0.0016	0.47 (0.26, 0.86)	0.0139
BMI, Median (Q1, Q3)	30.9 (26, 38.3)	31.8 (27.7, 38.8)	1.01 (0.98, 1.04)	0.4329		
Comorbidity, n (%)						
Cardiac Arrythmias	74 (48)	43 (59)	1.57 (0.89, 2.75)	0.1168		
CVD	23 (15)	16 (22)	1.61 (0.79, 3.28)	0.1878		
COPD	24 (15)	12 (16)	1.07 (0.5, 2.29)	0.8537		
CHF	21 (14)	21 (29)	2.58 (1.3, 5.11)	0.0067	1.65 (0.73, 3.72)	0.2293
Diabetes	41 (26)	22 (30)	1.2 (0.65, 2.22)	0.5618		
Hypertension	51 (33)	36 (49)	1.98 (1.12, 3.5)	0.0181	1.11 (0.56, 2.23)	0.7603
MI	15 (10)	10 (14)	1.48 (0.63, 3.48)	0.3668		
Obesity	23 (15)	15 (21)	1.48 (0.72, 3.05)	0.2823		
Renal Disease	23 (15)	14 (19)	1.36 (0.66, 2.83)	0.4082		
In-Hospital Characteristics						
ETT Size, n (%)						
<7.5	16 (15)	6 (10)	Reference			
7.5	55 (52)	30 (52)	1.45 (0.51, 4.11)	0.4796		
>7.5	35 (33)	22 (38)	1.68 (0.57, 4.93)	0.3483		
ETT: Height Ratio	0.1 (0.1, 0.1)	0.1 (0.1, 0.1)	1.12 (0.8, 1.57)	0.5233		
Mech Vent Duration, Median (Q1, Q3)	9 (4, 20)	13.5 (6.5, 19.5)	1.01 (0.98, 1.03)	0.5971		
Time From Extubation to Swallow 1, When Applicable, Median (Q1, Q3)	1 (1, 2)	1 (1, 2)	1.19 (0.81, 1.76)	0.3752		

In univariable associations, increasing age and hypertension remained significantly associated with risk of dysphagia at the second evaluation, and Hispanic ethnicity was associated with decreased risk of dysphagia. Additionally, cerebrovascular disease (CVD) was also associated with increased odds of dysphagia at the second evaluation. Ventilation duration and ETT size did not have any significant association (Table 3). Similar to the results at initial evaluation, multivariable analysis showed that increasing age was associated with increased risk of dysphagia (OR (95% CI): 1.06 (1.02, 1.10)) and Hispanic ethnicity was associated with decreased risk of dysphagia (OR (95% CI): 0.34 (0.15, 0.81)). Results were qualitatively similar in the sensitivity analysis using only those with an SLP evaluation.

**Table 3:** Associations with Dysphagia at Time 2 6 patients are excluded from the analysis, unable to impute time 2 dysphagia status. *Per unit Increase for age (years), mech vent (days), and time to swallow 2 (days).  Per 0.01 unit increase for ETT: height ratio. **Calculated using univariable logistic regression using complete case analysis. ***Calculated using multivariable logistic regression using all variables presented in the column. BMI, body mass index; CVD, cardiovascular disease; COPD, chronic obstructive pulmonary disease; CHF, chronic heart failure; MI, myocardial infarction; ETT, endotracheal tube; mech vent: mechanical ventilation

Patient Characteristics	Median (Q1, Q3) No Dysphagia, N=191	Median (Q1, Q3) Dysphagia, N=31	Univariable Odds Ratio* (95% Confidence Interval)	P-Value**	Multivariable Odds Ratio* (95% Confidence Interval)	P-Value***
Age, Median (Q1, Q3)	56 (43, 67)	68 (59, 78)	1.07 (1.03, 1.11)	< .0001>	1.06 (1.02, 1.10)	0.0019
Sex, Male, n (%)	115 (60)	18 (58)	0.92 (0.42, 1.98)	0.8212		
Race, n (%)						
White	97 (51)	15 (48)	Reference			
Black	32 (17)	10 (32)	2.02 (0.83, 4.94)	0.1231		
Other	62 (32)	6 (19)	0.63 (0.23, 1.7)	0.3578		
Ethnicity, Hispanic, n (%)	119 (64)	10 (32)	0.27 (0.12, 0.61)	0.0016	0.34 (0.15, 0.81)	0.0140
BMI, Median (Q1, Q3)	31.4 (26.0, 38.3)	31.6 (26.6, 39.5)	1.01 (0.97, 1.05)	0.5767		
Comorbidity, n (%)						
Cardiac Arrythmias	95 (50)	17 (55)	1.23 (0.57, 2.63)	0.5987		
CVD	28 (15)	11 (35)	3.2 (1.39, 7.4)	0.0065	1.25 (0.46, 3.38)	0.6657
COPD	32 (17)	3 (10)	0.53 (0.15, 1.86)	0.3228		
CHF	32 (17)	9 (29)	2.03 (0.86, 4.82)	0.1074		
Diabetes	47 (25)	12 (39)	1.94 (0.87, 4.28)	0.1033		
Hypertension	65 (34)	19 (61)	3.07 (1.4, 6.71)	0.0050	1.57 (0.62, 3.97)	0.3387
MI	20 (10)	5 (16)	1.64 (0.57, 4.76)	0.3593		
Obesity	30 (16)	7 (23)	1.57 (0.62, 3.96)	0.3439		
Renal Disease	29 (15)	8 (26)	1.94 (0.79, 4.76)	0.1464		
In-Hospital Characteristics						
ETT Size, n (%)						
<7.5	19 (14)	3 (13)	Reference			
7.5	71 (53)	10 (42)	0.89 (0.22, 3.57)	0.8716		
>7.5	45 (33)	11 (46)	1.55 (0.39, 6.18)	0.5361		
ETT: Height Ratio	0.1 (0.1, 0.1)	0.1 (0.1, 0.1)	0.96 (0.6, 1.51)	0.8479		
Mech Vent Duration, Median (Q1, Q3)	11 (5, 19)	12.5 (6, 20)	1 (0.97, 1.04)	0.9320		
Time From Extubation to Swallow 2, When Applicable, Median (Q1, Q3)	7 (5, 12)	9 (7, 12)	0.98 (0.93, 1.02)	0.3427		
Time From Swallow 1 to Swallow 2, When Applicable, Median (Q1, Q3)	6 (3, 11)	6 (4, 9)	1 (0.95, 1.05)	0.9015		

In the subset of patients with two SLP assessments, assessment of the pattern of progression between the first and last SLP evaluation demonstrated 55 of 73 patients to have an increase in score, indicating improvement in symptoms. Fifteen had a stable score, and three patients had a decrease in score. No demographic or in-hospital interventions were associated with a risk for worsening dysphagia over time. Mechanical ventilation duration was not associated with the progression of dysphagia over time.

## Discussion

Dysphagia is defined as an inability to safely transfer a bolus from the oral cavity to the stomach. It is very commonly associated with critically ill patients intubated for >48 hours and can have significant implications on a patient’s overall health status and prognosis [5]. Frajkova et al. list multiple mechanisms that contribute to the development of dysphagia after OTI, including laryngeal and oropharyngeal trauma, neuromuscular weakness, altered sensorium, and impaired synchronization of breathing and swallowing [4]. During the early SARS-CoV-2 pandemic, patients were intubated for lengths of time far outside the standard accepted timeframes. This was mainly due to the risk of tracheostomy on healthcare workers, as well as the poor prognosis of the critically ill Covid-19 patient. McGrath and colleagues discuss the worldwide ethical dilemma surrounding tracheostomy in Covid-19 patients, citing studies that indicate fewer than 12% of critically ill patients with tracheostomy are at home and functionally independent at one year [11,12]. For this reason, many proposed that the risk to healthcare workers did not outweigh the overall benefits to the patient. The atypical duration of intubation in this population allowed for a novel investigation into its effects. Additionally, given that all patients in our study were intubated due to Covid-19 critical illness and not from the typical myriad processes in an ICU setting, we were able to lessen other variables associated with intubation and take a more focused look at the impact of intubation duration on dysphagia.

In our ICUs, extubated patients received a bedside swallow screening by the nurse, which involves an assessment of patient general readiness for oral intake followed by a screening with small sips of water. If the patient has any signs of concern, such as choking, wet voice, or oral incompetence, a formal evaluation by SLP is done. Given that the SLP exam is bedside and requires relatively few hospital resources, it is a critical component in identifying dysphagia in patients who may not be stable enough to leave the unit for additional testing, like videofluoroscopy. 

Various systematic reviews and meta-analyses have been conducted to study the relationship between dysphagia and endotracheal intubation. Prominent in the literature are the findings of systematic reviews and meta-analyses by McIntyre et al. and Skoretz et al. Most recently, McIntyre et al. found a significant paucity in the standardized recording of dysphagia in the literature. Due to this limitation, their analysis found no risk factors to have a significant impact on the development of post-extubation dysphagia [13]. Despite the update and addition of further studies, their findings do not provide much more clarity when compared to those of Skoretz et al. in 2010, who stated that study quality and homogeneity greatly interfered with identifying a link between prolonged intubation, medical comorbidities, and the development of dysphagia [14]. 

Our study revealed that age impacted dysphagia at both the first and second SLP evaluations in univariable and multivariable analyses. This is consistent with other studies reported in the literature [15-17]. The impact was even stronger during the second evaluation, but there is no consensus in the literature on age and its impact on dysphagia.

Various studies have identified a link between the length of intubation and dysphagia, while others have found no association [5,6,8,15,17-22]. Our statistical analyses found no significance between the length of mechanical ventilation and the development of dysphagia at initial and subsequent evaluations. Additionally, the median length of intubation in the 134 patients not evaluated by SLP and imputed as having no dysphagia was identical to the length of intubation in our dysphagic population.

It was somewhat surprising to find, and important to note, that larger ETT size and ETT-to-height ratio did not have a direct correlation with dysphagia. However, this does not have any implications of the effects these variables have on voice and other laryngeal pathology and thus should not be misrepresented as an endorsement of using inappropriately large ETTs.

Among our population at a tertiary academic medical center, we found that CVD, hypertension, and diabetes were associated with an increased risk for dysphagia in univariable analyses. Ferraris et al. found similar findings in linking diabetes mellitus to dysphagia, but medical comorbidities have not been found to have a statistical link to dysphagia in post-intubation populations [14,16]. Interestingly, as noted in Table 3, our study found Hispanic individuals to be more likely to be in the non-dysphagia group. A review of the literature did not yield any findings investigating the effects of ethnicity on post-intubation dysphagia. It is possible that a language barrier or other factors may result in Hispanic patients being less likely to receive an SLP evaluation or communicate difficulty swallowing. This finding merits further investigation.

Compared to the population without an SLP evaluation, patients evaluated by SLP had a higher incidence of medical comorbid conditions. Our findings perhaps indicate that a patient’s comorbid factors are more crucial to the onset and continued symptoms of dysphagia than iatrogenic factors like ETT size and ventilation duration.

Most of our patients showed an improvement in dysphagia over time, as supported by the findings of Lindh et al., who showed an improvement in the FOIS score from extubation to hospital discharge. Their findings indicate that despite longer intubation duration, there is a relatively rapid return to swallowing in the critically ill Covid-19 population [23]. No demographic or in-hospital factors in our patient cohort were associated with a risk for worsening dysphagia over time. Duration of mechanical ventilation had no link to the progression of dysphagia with time. 

Our study has a few limitations. First, this is a retrospective study without a standardized use of fiberoptic endoscopic evaluation of swallowing (FEES) or video fluoroscopic swallow study (VFSS) for dysphagia evaluation. Our study uses the hospital protocol for swallow function post-extubation, which relies on the nurse and the ICU team to determine the need for further evaluation by SLP after a swallow screening. Additionally, the SLP evaluation is not necessarily inclusive of objective exams like videofluoroscopy. This could result in patients with “silent” dysphagia and even aspiration going undiagnosed. These exams are not only aerosol-generating procedures (AGPs) but are also more time intensive. During the pandemic, avoidance of AGPs and problems with staff shortages have unfortunately become key issues affecting patient assessment at many institutions, including ours. This may result in subclinical dysphagia going unnoticed. On the same note, the time between the SLP evaluations and the total number of SLP evaluations were not standardized and were customized based on each patient’s needs. Thus, generalizing findings across time may be difficult. Some of the patients with the longest durations of intubation underwent tracheostomies. This inserts a second variable into the dysphagia analysis but does provide a realistic and accurate incidence of dysphagia in the critically ill population. Our study is limited to a sample size of 308 patients with 94 patients receiving a swallow evaluation by SLP. It is possible that further associations may arise with larger sample sizes. 

Finally, we did not evaluate the patients for other long-term complications that are often associated with prolonged intubation. Voice changes and laryngeal and subglottic stenosis are crucial reasons to minimize the duration of OTI. Thus, this study does not infer that prolonged intubation complications are limited to dysphagia.

## Conclusions

Our findings show that although patient factors like advanced age, hypertension, CVD, and congestive heart failure were associated with dysphagia, intubation duration in the Covid-19 population does not significantly affect the development of dysphagia. Although dysphagia may not be a significant long-term sequala of prolonged intubation in the critically ill Covid-19 population, other laryngotracheal effects such as subglottic stenosis, granulomas, and laryngeal webbing continue to be a concern in all prolonged intubation population. Thus, the findings of this study should be interpreted with caution regarding the timing of tracheostomy, and a shorter duration of laryngotracheal intubation is still recommended. Further studies on the Covid-19 population with prolonged intubation would be of benefit in further elucidating these other sequelae.
